# Efficacy and safety of acupuncture in the treatment of foot drop in post-stroke: A protocol for systematic review and meta-analysis

**DOI:** 10.1097/MD.0000000000030994

**Published:** 2022-10-07

**Authors:** Ying Gao, Xiaochao Gang, Yue Yuan, Kai Yin, Xiaoyan Gong

**Affiliations:** a Doctor of Medicine, School of Traditional Chinese Medicine, Changchun University of Chinese Medicine, Changchun, China; b Doctor of Medicine, College of acupuncture and massage, Changchun University of Chinese Medicine, Changchun, China; c Doctor of Medicine, School of Basic Medicine, Changchun University of Chinese Medicine, Changchun, China; d Master of Medicine, School of Rehabilitation Medicine, Changchun University of Chinese Medicine, Changchun, China; e Master of Medicine, The Affiliated Hospital of Changchun University of Chinese Medicine, Changchun, China.

**Keywords:** acupuncture, foot drop in post-stroke, protocol, systematic review

## Abstract

Foot drop is a common complication in post-stroke. Patients with foot drop are at high risk for falls and fall-related injuries. Accordingly, it can reduce independence and quality of life in patients. Clinical studies have confirmed that acupuncture is effective in treating foot drop in post-stroke. However, there is a lack of systematic review exploring the efficacy and safety of acupuncture treatment. This study aims to assess the efficacy and safety of acupuncture in the treatment of foot drop in poststroke from the results of randomized controlled trials.

**Methods:**

We will search articles in 8 electronic databases including the Cochrane Central Register of Controlled Trials, the Web of Science, PubMed, Embase, the China National Knowledge Infrastructure, the Chinese Biomedical Literature Database, Wanfang Data Database, and the Chinese Scientific Journal Database for RCTs of acupuncture treated foot drop in post-stroke from their inception to 10 August 2022. We will analyze the data meeting the inclusion criteria with the RevMan V.5.4 software. Two authors will assess the quality of the study with the Cochrane collaborative risk bias tool. We will evaluate the certainty of the estimated evidence with the Grading of Recommendations Assessment, Development, and Evaluation (GRADE) method. Data analysis will be performed using STATA 16.0.

**Results:**

This study will review and evaluate the available evidence for the treatment of foot drop in post-stroke using acupuncture.

**Conclusion subsections:**

This study will determine the efficacy and safety of acupuncture applied to post-stroke individuals with foot drop.

## 1. Introduction

Stroke is a common clinical cerebrovascular disease, including hemorrhagic stroke and ischemic stroke. In recent years, the incidence of stroke has been raised, and it has become the main cause of mortality and disability in the elderly.^[1]^ Surviving patients are often left with limb dysfunction whom more than 80%,^[2]^ and foot drop is one of the common symptoms.^[3]^ As estimated, the incidence rate of foot drop after stroke is as high as 20% to 30%.^[4,5]^ This condition is related to the weakness or lack of voluntary control in ankle dorsiflexors and/or the increased spasticity of plantar flexor muscles.^[6–8]^ Foot drop results in disruption of weight acceptance and weight transfer in the initial foot contact and stance phases and interferes with ankle dorsiflexion during the swing phase of the gait.^[9]^ Thus, foot drop leads to an inefficient walking speed, reduces gait stability,^[10]^ and increases the risk of falls.^[11]^ In this condition, gait impairment may restrict activities of daily living, affect independence, and reduce the quality of life.^[12]^ Therefore, it is important to treat foot drop to ensure a safe gait for stroke patients.

Rehabilitation and physical therapy, drug therapy, and operation therapy are the primary methods in Western medicine to treat foot drop in post-stroke. However, drug treatment has large side effects which hinder the recovery of motor function with long time taking.^[13]^ Two types of physical therapy commonly used today are the installation of an ankle-foot orthosis (AFO) or the use of functional electrical stimulation (FES) via an electronic stimulator. The previous study has shown that AFO can improve gait velocity, cadence, and stride length.^[14]^ But AFO only has an immediate orthotic effect, so the foot drop reoccurs without it. FES is applied to the common peroneal nerve, causing contraction of the tibialis anterior muscle and ankle dorsiflexion, to reduce foot drop.^[15]^ But it has limitations on patient compliance, adaptation, and comfort. As for surgical treatment, most patients cannot accept it because it is traumatic.

Acupuncture is a unique external treatment method of traditional Chinese medicine, which has a history of thousands of years. Acupuncture stimulates acupoints on the body surface, transmits information to the central nervous system, and then stimulates motor nerve cells from the central nervous system, which can stimulate the antagonistic muscle groups and inhibit the spasmodic muscle groups, thereby improving the body’s motor function. Some clinical studies have confirmed the advantages of acupuncture in the treatment of hemiplegic, which can improve the gait of the foot drop in post-stroke.^[16,17]^ However, there is currently no systematic review exploring the efficacy of acupuncture treatment. So this study will collect data on randomized controlled trials (RCTs) of acupuncture for foot drop in post-stroke. These data will be synthesized in a systematic review with meta-analysis to explore the efficacy and safety of acupuncture in the treatment of foot drop in post-stroke, and to provide evidence for clinical decision-making of acupuncture.

## 2. Methods and analysis

### 2.1. Design and registration of the review

The protocol has been registered on PROSPERO basing on the Preferred Reporting Items for Systematic Reviews and Meta-Analyses Protocols (PRISMA-P) statement guidelines.^[18]^ The registration number is CRD42022325951.

### 2.2. Inclusion criteria

#### 2.2.1. Types of studies.

We will collect RCTs (not including quasi-RCTs) of acupuncture in the treatment of foot drop in post-stroke. All eligible trials will be included regardless of language and publication type. Review articles, case series, cohort studies, retrospective studies, and animal experiments that do not meet the requirements will be excluded.

#### 2.2.2. Types of patients.

Patients must meet the following requirements: In line with “2016 Guidelines and Consensus on Diagnosis and Treatment of Chinese Cerebrovascular Diseases”, and were first confirmed by cerebral CT or MRI as having a stroke (cerebral infarction or cerebral hemorrhage); age > 18 years old, and disease course < 6 months; have the symptoms of hemiplegia in one limb and foot drop; the Modified Ashworth Scale (MAS) score is grade 1 to 4.

#### 2.2.3. Types of interventions.

In the intervention group, patients received acupuncture, alone or combined with routine rehabilitation treatment (FES, ankle-foot orthoses, and physiotherapy, among others). In the control group, patients only received routine rehabilitation treatment.

#### 2.2.4. Types of outcome measures.

##### 2.2.4.1. Primary outcomes.

- Gait speed will be assessed by the 10-m Walk Test (10MWT).It will be calculated as the ratio between distance and time spent (seconds) to walk 10 m.
- Spasticity of plantar flexor muscles will be assessed by the MAS.

##### 2.2.5.1. Secondary outcomes.

- Anterior tibial muscle strength will be assessed by the Modified Lovett test;
- Lower extremity motor function will be assessed by the Fugl-Meyer Assessment Scale (FMA);
- Daily living ability will be assessed by the Modified Barthel Index;
- The improvement of foot drop will be assessed by the Garceau standard.

### 2.3. Exclusion criteria

Studies with the following situations will be excluded: foot drop due to a neurological diagnosis other than stroke; the patient has lower limb fractures; studies comparing the same modality of acupuncture with different doses of time, frequency, or duration; conference abstracts without a full published article; duplicated data or data that cannot be extracted.

### 2.4. Search methods for the identifying of studies

Two reviewers (YG and XG) will independently search the China National Knowledge Infrastructure, the Chinese Biomedical Literature Database, Wanfang Data, the Chinese Scientific Journal Database, the Cochrane Central Register of Controlled Trials, the Web of Science, PubMed, and Embase databases from inception to 10 August 2022. Except for electronic search, we will also hand search the conference summary and trial register. Any disagreement will be resolved by discussion until consensus is reached or by consulting a third researcher (YY).

### 2.5. Search strategy

The search strategy will be based on the Cochrane handbook guidelines (V.5.1.0) including keywords such as “post-stroke”, “foot drop” or “hemiplegia”, “acupuncture”, and “RCT”. Subsequent searches will use Medical Subject Headings (MeSH) headings, including “post-stroke”, and “acupuncture”, in addition to keywords from the initial retrieval. The reference lists of relevant research articles were reviewed for any additional article searches. The details of the PubMed search strategies are provided in Table 1. Other electronic databases are also suitable for this search strategy.

**Table 1:** The search strategy for Pub Med database.

No	Search terms
#1	post-stroke.ti,mesh.
#2	poststroke.ti,ab.
#3	Apoplectic.ti,ab.
#4	after stroke.ti,ab.
#5	Cerebral hemorrhage. ti,ab.
#6	Cerebral embolism
#7	#1 or #2 or #3 or #4 or #5 or #6
#8	acupuncture.ti,mesh.
#9	acupoint.ti,ab.
#10	fire-needle.ti,ab.
#11	three-edged needle.ti,ab.
#12	plum blossom needle.ti,ab.
#13	pinprick.ti,ab.
#14	needle therapy.ti,ab.
#15	needle.ti,ab.
#16	#8 or #9 or #10 or #11 or #12 or #13 or #14 or #15
#17	randomized controlled trial.pt.
#18	randomized.ab.
#19	trial.ab.
#20	#17 or #18 or #19
#21	#7 and #16 and #20

### 2.6. Data extraction

#### 2.6.1. Study selection.

**Figure 1.**
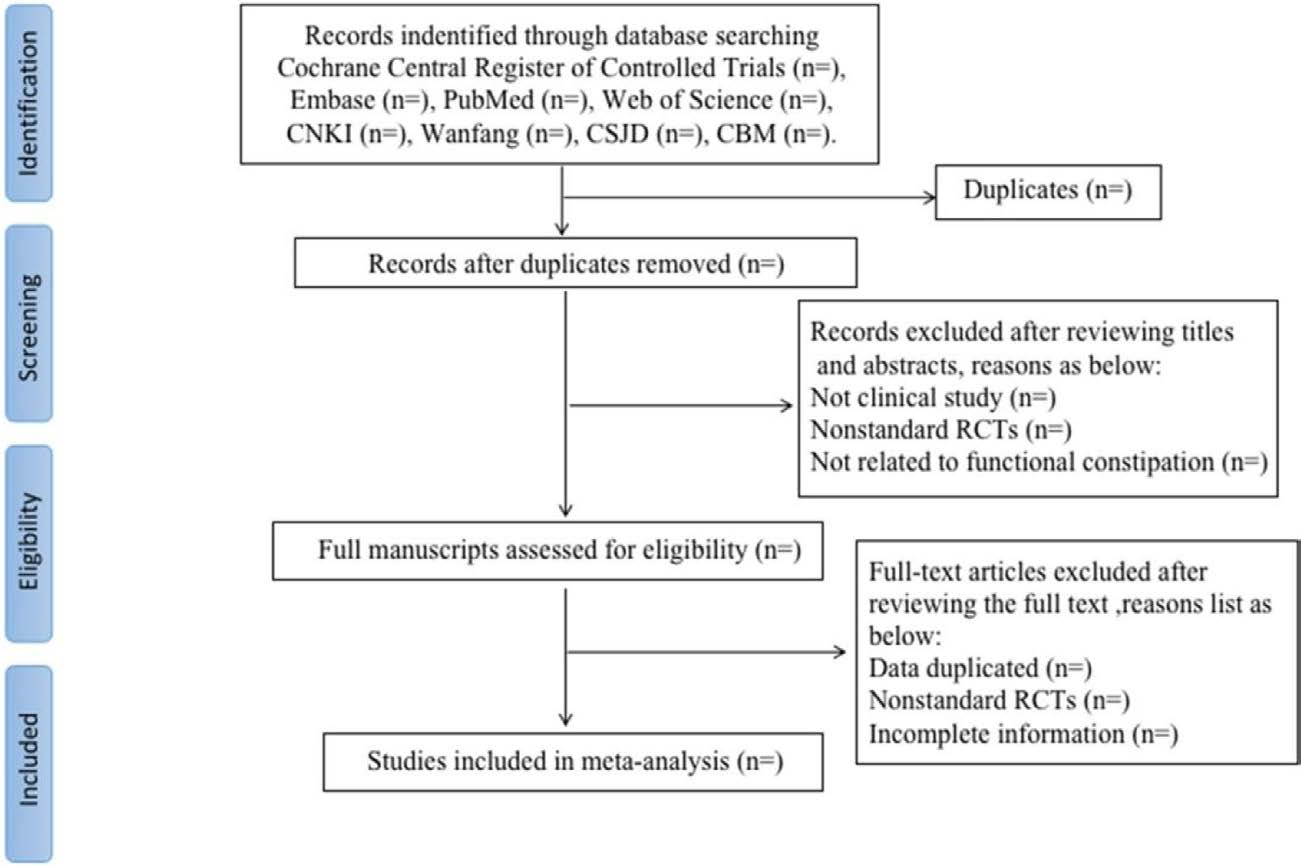
Flow chart of the search process.

Records from databases and other resources will be uploaded to a database created in EndNote software(X.9.3.3). Two review authors (YG and XG) will independently evaluate the titles and abstracts of all citations found from the above search strategy. We will obtain the full text of all potentially suitable articles to further assess eligibility based on the inclusion/exclusion criteria. The disagreement will be resolved by consensus or mediated by a third author (YY). The final selection process will follow the PRISMA guidelines,^[18]^as shown in Figure 1.

#### 2.6.2. Data extraction and management.

Two reviewers (YG and XG) will independently apply the inclusion and exclusion criteria to assess each retrieved study’s eligibility. The data will then be extracted from the selected studies for inclusion using a data collection form and recorded in an Excel file. The following data will be extracted: study characteristics such as author, year of publication, country where the study was conducted, study period, original inclusion criteria, total number of people included in the study; population characteristics such as type of stroke, mean age, and time from diagnosis; intervention characteristics such as type, duration, and frequency; outcomes such as the 10MWT, MAS score, FMA, Modified Lovett test, Modified Barthel Index, and the Garceau standard. Disagreements in data collection will be resolved by consensus, with any ongoing differences in opinion arbitrated by a third reviewer (YY). If possible, we will contact the author of the article to provide additional relevant information.

### 2.7. Risk of bias assessment

Two review authors (YG and XG) will assess the internal validity of included studies by using the Cochrane Handbook for Systematic Reviews of Interventions.^[19]^ The following domains will be assessed: random sequence generation and allocation concealment (selection bias), blinding of participants and personnel (performance bias), incomplete outcome data (attrition bias), outcome assessment (detection bias), selection of the reported result (reporting bias), and other biases (such as pre-sample size estimation, early stop of trial). Results from these questions will be graphed and assessed using Review Manager 5.4. Each item will be evaluated in three categories: low risk of bias, unclear bias, and high risk of bias. Any discrepancies will be resolved by reviewing the original article and discussing it with the third author (YY) to reach a consensus.

### 2.8. Measures of treatment effect

Data will be subjected to statistical analysis using RevMan5.4 software. For continuous variables, we will present mean differences with standard deviations, or median and interquartile range. For dichotomous variables, we will report the overall mean proportion (%) of the population. The 95% CI will be presented for both dichotomous and continuous outcomes.

#### 2.8.1. Missing data management.

We will obtain the missing data by contacting the original authors of the papers via email or telephone if possible. Otherwise, we will analyze the available information and conduct a sensitivity analysis to explore the potential impact of insufficient information on the results of the meta-analysis.

### 2.9. Heterogeneity assessment

Statistical heterogeneity will be assessed with the *I*^2^ statistic. We will interpret it using the following criteria: *I*^2^ values of 25% is considered low levels of heterogeneity, 50% indicated moderate levels, and 75% indicated high levels.^[20]^ Since low or moderate heterogeneity suggests little variability among these studies, the data will be analyzed in a fixed-effects model.^[21]^ When significant heterogeneity occurs among the studies (*P* < .05, *I*^2^ > 50%), a random-effect model will be performed to analyze the data.

### 2.10. Assessment of reporting biases

Reporting bias will be assessed by Egger’s regression asymmetry test.^[22]^ A *P* value < .05 in the Egger test is considered statistically significant. STATA V.16.0.^b^ software will be used to perform Egger test.

### 2.11. Subgroup analysis

We plan to carry out the following subgroup analyses to explore possible sources of heterogeneity: different types of stroke, the course of the disease, different types of acupuncture, different times of treatment, and different types of routine rehabilitation methods. If the data is insufficient, the qualitative synthesis will be conducted instead of the quantitative synthesis. We will use the formal test for subgroup interactions in Review Manager V.5.4.

### 2.12. Sensitivity analysis

When possible, we will perform sensitivity analysis by eliminating low-quality trials to explore the effects of the trial’s bias risk on primary outcomes.

### 2.13. Grading the quality of evidence

The quality of evidence regarding patient outcomes will be used and assessed by the Grading of Recommendations Assessment, Development, and Evaluation methodology (GRADE; https://www.Gradeworkinggroup.org/). GRADE will be used to summarize the limitations in design, consistency, directness, precision, and publication bias. The quality of each piece of evidence will be divided into four levels: high, medium, low, and very low.

## 3. Ethics and dissemination

This systemic review will evaluate the efficacy and safety of acupuncture in the treatment of foot drop in post-stroke. Since all included data will be obtained from published articles, it does not require ethical approval and will be published in a peer-reviewed journal. The results may contribute to improving the therapeutic strategy of patients with foot drop in post-stroke.

## 4. Discussion

Foot drop after stroke is a relatively common type of sequelae that is mainly caused by central nervous system injury.^[23]^ The patient tends to an abnormal posture such as “circling gait” due to foot drop, which will hinder the establishment of normal mobility mode and affect the recovery of lower limb motor function in hemiplegia.^[24]^ Acupuncture can stimulate the meridians to improve local blood circulation in the brain tissue of stroke patients and improve neurological deficits. And acupuncture also can eliminate contractures of the posterior calf muscles, improve the muscle group tension of the posterior calf and achieve the effect of treating foot drop.^[25,26]^ By acupuncture Indwelling the traction stimulation for soft tissue, the contraction force of muscle fibers is enhanced, and unidirectionally pulling the front calf muscle group strongly, which can achieve the effect of straightening. Compared with rehabilitation training, acupuncture has the characteristics of convenient operation, low cost, and a wide range of indications. Some studies show acupuncture is good at treating foot drop and has been clinically verified. However, from the existing literature, and considering the lack of meta-analyses exploring the effects of acupuncture, we cannot make evidence-based inferences about the clinical benefit of acupuncture for foot drop in post-stroke. This review aims to determine the efficacy and safety of acupuncture applied to post-stroke individuals with foot drop. We will analyze the effect of acupuncture combined or not with other therapies on gait speed, muscle strength, lower extremity motor function, daily living ability, and the improvement of foot drop.

## Author contributions

**Conceptualization:** Ying Gao.

**Formal analysis:** Ying Gao, Xiaochao Gang.

**Investigation:** Ying Gao, Xiaochao Gang.

**Methodology:** Xiaochao Gang, Yue Yuan.

**Supervision:** Ying Gao, Xiao Yan Gong.

**Writing – original draft:** Ying Gao, Xiaochao Gang, Yue Yuan, Kai Yin.

**Writing – review &amp; editing:** Xiao Yan Gong.

**Funding acquisition:** Xiaochao Gang.

**Data curation:** Yue Yuan, Kai Yin.

**Software:** Kai Yin.
